# Synthesis of Imidazole-Based Deep Eutectic Solvents as Solid Lubricants: Lubricated State Transition

**DOI:** 10.3390/ma16196579

**Published:** 2023-10-06

**Authors:** Houjie Zhang, Youming Chen, Aimin Chu, Hairong Hu, Yuping Zhao

**Affiliations:** 1 Health Maintenance for Mechanical Equipment Key Lab of Hunan Province, Hunan University of Science and Technology, Xiangtan 411201, China; 2 School of Materials Science and Engineering, Hunan University of Science and Technology, Xiangtan 411201, China; 3 School of Civil and Engineering, Hunan University of Science and Technology, Xiangtan 411201, China

**Keywords:** deep eutectic solvents, solid lubricants, lubricated state transition, imidazole

## Abstract

The controllable character of the melting point of deep eutectic solvents (DESs) makes it easy to realize lubricated state transitions and produce excellent lubricating properties during friction. In this work, a series of novel imidazole-based DESs were synthesized to present a room-temperature solid state by shifting its eutectic point. Tribological test results show that the wear volume of these DESs decreases as the alkyl chains of the hydrogen bond donors increase. A proper deviation of the eutectic point in DESs produces stable lubricating properties. The present work provides a novel and simple method to prepare solid lubricants and enriches the use of DESs as lubricants. Simultaneously, the method expected to replace the use of conventional cutting fluids.

## 1. Introduction

During the mechanical machining process, cutting fluids are supplied continuously to the corresponding contact zone to reduce friction. Cutting fluid has a certain service life, and if mixed with metal chips, dust, machine lubricants, engine oil, and other impurities, will show emulsion deterioration and cutting fluid waste formation [1,2,3]. In addition, they are often considered dangerous to workers’ health and the environment due to their synthetic nature and storage problems [4,5]. Solid lubricants are considered environmentally friendly, biodegradable, and inherently safe, but the thickness of solid lubricants is limited and they will eventually be worn, degrading their lubricating properties [6,7,8]. Multiple methods have been tried by researchers to combine the advantages in developing new lubricating materials [9,10]. However, the use of dual-phase behavior to design cutting fluids has rarely been noticed by researchers [11].

Deep eutectic solvents (DESs), as a system consisting of hydrogen bond donors and hydrogen bond acceptors, were reported by Abbott et al. in 2001 [12]. They discovered some eutectic mixtures based on urea and a range of quaternary ammonium salts to form low-melting-point eutectic crystals with unusual solvent properties [13]. Like ionic liquids (ILs), DESs have the characteristics of being non-volatile, non-flammable, polar, and degradable, and a high utilization rate [14]. In addition, they are characterized by the fact that the melting point of the combined system is lower than its pure component, so it has the characteristics of an adjustable melting point and adjustable chemical structure [15,16,17,18]. Therefore, it is possible to use DESs as lubricating materials and realize the transition of lubrication due to its characteristics.

Pure lubricants and the addition of base oils were considered the most common strategies of DESs in the field of lubrication. The first to apply DESs to the field of friction was Lawes et al. The DESs of choline chloride in combination with ethylamine and urea were applied by them in steel-to-steel friction [19]. The lubricating properties of choline-based DESs were compared with those of imidazolyl-based ILs, firstly with the former showing improved wear on iron-based alloys [20]. In 2019, the friction coefficient of DESs with sulfur-based ILs as hydrogen bond acceptors was reduced to 0.1 in lubrication tests with silicon surfaces [21]. In later work, the effect of the molar ratio of such DESs on lubrication properties was explored, and the best results were attributed to the strong hydrogen bonding [22]. The lubricating properties of the two DESs with different physical states were analyzed in terms of fretting and sliding [23]. On the other hand, there is also more and more research on DESs as additives for base oils. Khan et al. used an aminoguanidine salt-based DES as an additive for mineral oil, and DES-derived lubrication and the protective film improved friction [24]. Recently, an oil-soluble deep eutectic solvent as a high-performance green lubricant additive for PAO 40 and PEG 200 [25] was used. Although DESs are increasingly reported in pure lubricants and base oil additives, the advantage of the melting point adjustability of DESs is not used by researchers.

Based on the above research, it is necessary to improve the lubrication performance of DESs under the condition of ensuring that their lubricating state changes. The latest trend in the area of tribology is the investigation of ILs as an alternative lubricant for its structural adjustability [26]. Imidazole ILs are currently the most widely used, and the longer the alkyl group, the better the adsorption capacity and the better the friction and anti-wear properties of ionic liquid [27,28]. In addition, the lubricating properties of DESs may be related to the alkyl chain length of the hydrogen bond donor. In this work, 1-butyl-3-methylimidazolium chloride salt was used as the hydrogen bond acceptor, and an amide with different chain lengths was used as the hydrogen bond donor. A series of solid DESs were synthesized via deviation from the eutectic point. The influence of hydrogen bond donors on lubricating properties was investigated in the steel-to-steel friction pair. The surface topography and chemical composition of the friction marks were emphasized, and the friction mechanism that may have occurred was discussed.

## 2. Experiment Details

### 2.1. Materials

1-Butyl-3-methylimidazolium chloride (BmimCl, pure > 98%), acetamide (pure > 99%), propionamide (pure > 96%), and butyramide (pure > 98%) were purchased from Aladdin Biochemical Technology Co., Ltd. (Shanghai, China). All the chemicals were used as received without further purification. All materials were dried under vacuum at 60 °C for two days before use.

### 2.2. DESs Preparation

The 1-butyl-3-methylimidazolium chloride salt was chosen as the hydrogen bond acceptor and an amide was chosen as the hydrogen bond donor for DES preparation. The mixtures of salt and amide were heated and stirred at 80–100 °C to form a homogeneous liquid. The molar ratio of BmimCl to hydrogen bond donors was chosen to be 1:4 for their crystallization. Table 1 shows the structure of DESs and the molar ratio of the hydrogen bond acceptor to the hydrogen bond donors.

### 2.3. Friction Test

The reciprocating friction test is a common tool used for testing lubricants, and steel is the most commonly used material in mechanical manufacturing. The lubricating properties of all DESs as lubricants were investigated using a HSR-2M high-speed reciprocating friction and wear-testing machine with a ball-on-disk frictional pair. The upper stationary ball (ø 6 mm) was made of GCr15 bearing steel and the lower running disk (ø 25 mm × 8 mm) was made of 304 stainless steel. The friction and wear tests were conducted under conditions of 20 N, 600 RPM, 5 mm, and 30 min. Before each test, balls and steel discs were cleaned with ethanol in an ultrasonic bath. To guarantee the reliability of the results, each EDS was tested at least three times. After the friction test, the wear volume and 3D morphology of the lower test plate were measured using a three-dimensional profiler (NanoMap-500LS, Santa Clara, CA, USA).

### 2.4. Characterization

The microstructure of DESs was measured using a Fourier transform infrared spectrophotometer (FTIR, Nicolet 6700, Waltham, MA, USA) in the 4000–400 cm^−1^ range, and they were dissolved with ethanol before the test. The thermal properties of the DESs were analyzed vis differential scanning calorimetry (DSC, Netzsch200 F3, Selb, Germany). These samples were sealed in aluminum crucibles in an inert atmosphere. One cycle in the temperature range of −40 °C to 150 °C, with a cooling/heating velocity of 10 °C per minute, was carried out. The discs were cleaned after the friction test and the worn surfaces were characterized using a scanning electron microscope (SEM, TESCAN MIRA, Brno, Czech Republic) equipped with an energy-dispersive spectrometer (EDS). The chemical states of the typical elements on the worn surfaces were analyzed via X-ray photoelectron spectroscopy (XPS, THERMO Scientific K-Alpha, Waltham, MA, USA) with Al Kα as the excitation source.

## 3. Results and Discussion

### 3.1. Characterization of DESs

**Figure 1:**
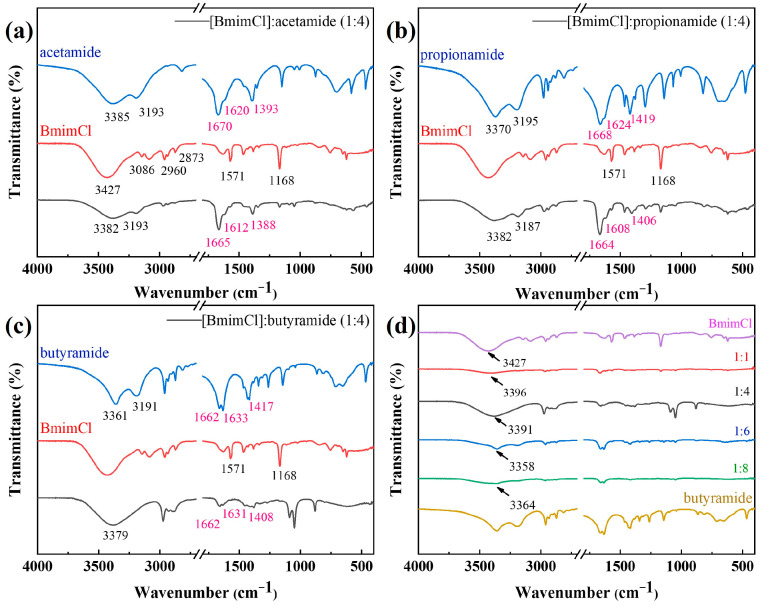
FTIR spectra of (**a**) [BmimCl]: acetamide (1:4), (**b**) [BmimCl]: propionamide (1:4), (**c**) [BmimCl]: butyramide (1:4), and (**d**) [BmimCl]: butyramide (1:1, 1:4, 1:6, and 1:8) in wavenumber ranges.

The FTIR spectra used to characterize the structure of all DESs are presented in Figure 1. In the infrared spectrum of pure BmimCl, the peak around 3427 cm^−1^ is attributed to moisture. The absorption peak around 3086 cm^−1^ was caused by the C-H stretching vibration on the imidazole cation. The absorption peak of the C-H stretching vibration of methyl and methylene groups is represented in the range of 2873–2960 cm^−1^. The source of the absorption peak at 1571 cm^−1^ is the vibration from the imidazole ring backbone and the stretching vibration of the imidazole ring at 1168 cm^−1^ [29]. In the infrared spectrum of pure amide, the two peaks around 3300 cm^−1^ are attributed to the N-H stretching vibration [30]. The strong signal of N-H deformation vibration peaks is found in 1620 cm^−1^. The peaks of the C-N stretching frequency are observed around 1410 cm^−1^. A clear signal of the carbonyl C atom (C=O) is found at 1660 cm^−1^.

By comparing the spectra obtained from the DESs with those of BmimCl and the amide, it is clear that the BmimCl and amide spectrum is an overlap of the other two spectra, but with slight differences. The synthesis of DESs can be verified via the shifts and widening in the representative peaks of the involved bonds in the FTIR spectra [31,32]. For the BmimCl–amide system, the stretching vibrations of C=O (1665 cm^−1^) and the bending vibration of N-H (1620 cm^−1^) in pure Amida were red-shifted to 1660 cm^−1^ and 1610 cm^−1^ in the DES system (Figure 1a–c), indicating the occurrence of intermolecular hydrogen bonding. In addition, a red-shift of the stretching vibrations of C-N occurred from approximately 1410 cm^−1^ to 1400 cm^−1^ in the DES system (Figure 1a–c). Specifically, an H bond was formed between the NH_2_ of Amida and Cl^−^ of BmimCl. Interestingly, the two peaks around 3300 cm^−1^ in pure Amida did not shift significantly in the DES system. One possible explanation is that these DESs were affected by water absorption. These above results suggest that the DESs were successfully synthesized.

A comparison of [BmimCl]: butyramide of different molar ratios is shown in Figure 1d at wavenumber ranges. It can be found that a red-shift was presented in the vibration peak at 3350 cm^−1^ as the proportion of the amide increased. This suggests that the chemical bonds increase between DESs along with the proportion of hydrogen bond donors. The hydrogen bond established between the imidazolium chloride salt and the amides should have induced longer bonds in the ions involved, reducing the bond strength and frequency, but the formation of new hydrogen bonds may have involved blue- and red-shifts, which are consistent with the electron density distribution, depending on the electron affinity of the atoms involved [33].

**Figure 2:**
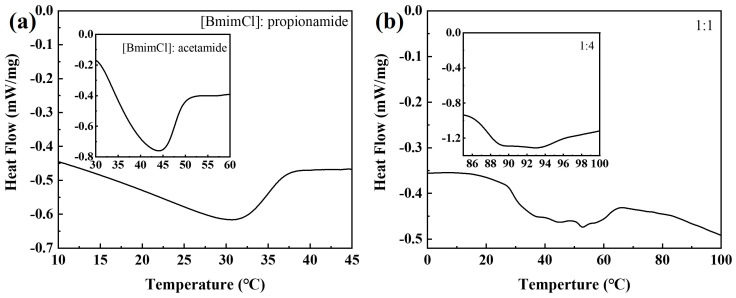
DSC of (**a**) [BmimCl]: acetamide, [BmimCl]: propionamide, and (**b**) [BmimCl]: butyramide (1:1, 1:4).

The melting point drop of DESs is another important sign of heir synthesis [34,35]. The DSC of [BmimCl]: acetamide, [BmimCl]: propionamide, and [BmimCl]: butyramide are presented in Figure 2 and Table 2. Figure 2a shows that the melting point peaks of [BmimCl]: acetamide and [BmimCl]: propionamide are 30.24 °C and 40.10 °C, respectively, which are lower than those of their pure components (BmimCl = 73 °C, acetamide = 81 °C, and propionamide = 114 °C). The reduction in the melting point is due to the interaction between the anion of the salt and the hydrogen bond and is influenced by the type of interaction, the entropy change accompanying the formation of the liquid phase, and the lattice energy of DESs [36,37]. Figure 2b shows that the melting point peaks of [BmimCl]: butyramide (1:1 and 1:4) are 44.19 °C and 93.01 °C, respectively. The melting points of these DESs were affected by the molar ratio between salt and hydrogen bond donors. This is attributed to the melting point adjustability of DESs [38]. Therefore, these DESs present solid and semi-solid forms at room temperature, which makes them have certain advantages in the field of lubrication. It is worth noting that the melting point of [BmimCl]: butyramide (1:4) is close to the melting point of its pure components, possibly because this composition deviates more from its eutectic point [39]. These results show that these solid DESs at room temperature were successfully synthesized via the regulation of their melting points.

### 3.2. Frictional Wear

**Figure 3:**
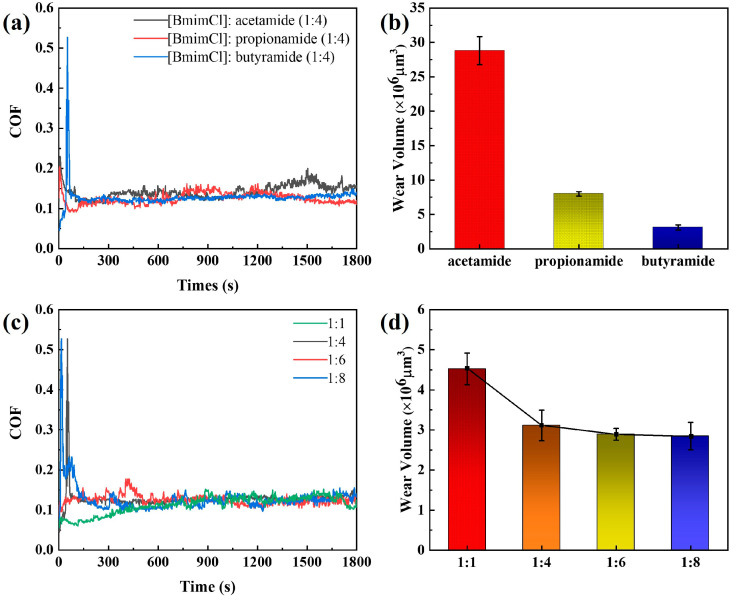
The COF and wear volume of DESs under conditions of 20 N, 600 RPM, 5 mm, and 30 min. (**a**,**b**) [BmimCl]: acetamide (1:4), [BmimCl]: propionamide (1:4), and [BmimCl]: butyramide (1:4); (**c**,**d**) [BmimCl]: butyramide (1:1, 1:4, 1:6, and 1:8).

The tribological behavior of these DESs was assessed via the friction coefficient and wear volumes in Figure 3. On the one hand, the friction coefficient of [BmimCl]: acetamide (1:4), [BmimCl]: propionamide (1:4), and [BmimCl]: butyramide (1:4) are shown in Figure 3a. Their friction coefficients can be believed to differ a little due to the fact that the trial was conducted more than three times. This suggests that the friction coefficient of these DESs were affected by BmimCl chiefly. Especially, the coefficient of friction curve of [BmimCl]: butyramide (1:4) appears to be more stable. The lubrication regime may be considered a boundary/mixed. On the other hand, the wear volumes of [BmimCl]: acetamide (1:4), [BmimCl]: propionamide (1:4), and [BmimCl]: butyramide (1:4) are shown in Figure 3b. The wear volume of the three DESs are around 30 × 10^6^ μm^3^, 8 × 10^6^ μm^3^, and 3 × 10^6^ μm^3^, respectively. The wear volume decreases with the increase in the chain length of the hydrogen bond donors. This suggests that the tribological behavior of DESs was affected by the alkyl chain length of hydrogen bond donors. This result is similar to that of IL lubrication. The advantage of the chain length is widely recognized by researchers in field of the boundary lubrication of ILs [40,41,42]. In addition, the friction coefficient of [BmimCl]: acetamide (1:4) is described as being in great swing compared to that of other DESs. The weakened lubrication can be attributed to their low melting point, which allows them to absorb water under friction [43,44,45].

To further understand the lubricating properties of these DESs, [BmimCl]: butyramide was taken to make a series of different proportions to test the tribological properties. The friction coefficient and wear volumes of these DESs are presented in Figure 3c,d. The coefficient of friction did not change significantly, even though the hydrogen bond donor contents of these DESs changed. This suggests that the lubricating properties of DESs are more dependent on the type of hydrogen bond acceptor. In other words, lubricating performance is not significantly improved by the content of hydrogen bond donors in the same DES. Interestingly, it should have better lubricating properties as the ratio of [BmimCl]: butyramide becomes closer to the eutectic point [15]. However, it was found that the [BmimCl]: butyramide (1:1) has a lower coefficient of friction in the anterior friction only. A possible explanation for this behavior may be attributed to solid DESs at room temperature. In addition, the largest wear volume was formulated in [BmimCl]: butyramide (1:1). This can also be attributed to the [BmimCl]: butyramide (1:1) closest to the eutectic point. This suggests that higher melting points make the lubricating properties of these DESs more stable. In conclusion, the friction test results show that these imidazole-based DESs have a stable coefficient of friction and low wear as the alkyl group of hydrogen bond donors increases.

### 3.3. Surface and Compositional Analysis

**Figure 4:**
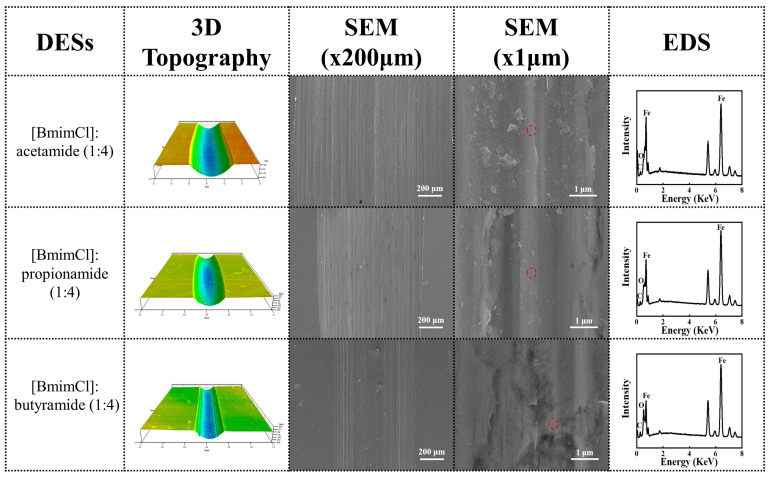
Figure showing 3D topography, SEM micrographs, and EDS of the scanned spot.

The 3D diagrams and SEM images of DESs comparing amides with different alkyl chain lengths are shown in Figure 4. It can be seen that [BmimCl]: acetamide (1:4) showed the largest wear area, [BmimCl]: propionamide (1:4) showed the second-largest wear area, and [BmimCl]: butyramide (1:4) had the smallest wear profile as determined via 3D topography. The results are consistent with the wear volume law of the previous section and the SEM images of the worn surfaces under SEM images (×200 μm). Furthermore, many fine particles can be seen on the worn surfaces of [BmimCl]: acetamide (1:4), [BmimCl]: propionamide (1:4), and [BmimCl]: butyramide (1:4) in the SEM images (×200 μm). The particles can be seen more clearly in high-resolution SEM images (×1 μm). This suggests that abrasive wear occurs on the surface of steel under the lubrication of [BmimCl]: acetamide (1:4), [BmimCl]: propionamide (1:4), and [BmimCl]: butyramide (1:4) [46]. In addition, the most abrasive particles were found on the worn surface lubricated with [BmimCl]: acetamide under the same magnification (×1 μm), representing the most serious wear. On the contrary, the fewest abrasive particles were found on the worn surface lubricated wit [BmimCl]: butyramide, representing minimal wear. The results show that these DESs make abrasive grains play a large role in the process of steel-to-steel friction.

**Figure 5:**
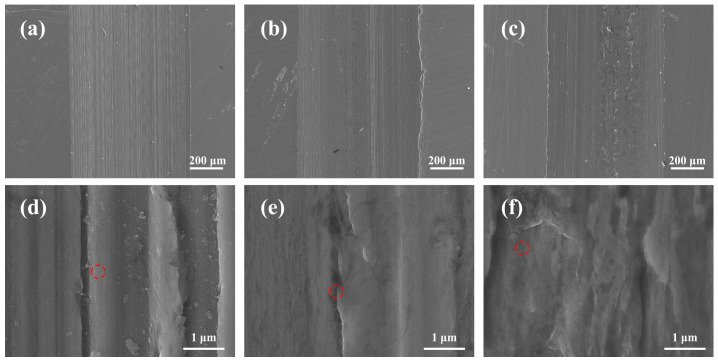
SEM images (×200 μm and ×1 μm): (**a**,**d**) [BmimCl]: butyramide (1:1), (**b**,**e**) [BmimCl]: butyramide (1:6), and (**c**,**f**) [BmimCl]: butyramide (1:8).

There were useful C, O, and Fe elements found via EDS analysis from the scanned spot on a smooth surface (Figure 4 and Figure 5). The formation of tribo-chemical films was disturbed by the elements of the substrate itself due to the detected depth of the EDS. Intact point scan for all DESs are shown in Table 3. A reasonable guess is the lubricating film being involved in the C, O, and Fe elements produced on the worn surface. Interestingly, there was also an increase of O element from [BmimCl]: acetamide to [BmimCl]: butyramide on the wear scar, which may have been due to the richer oxide film [47]. However, further surface analysis is necessary to prove this proposed explanation.

The SEM images of [BmimCl]: butyramide are shown in Figure 5. It can be found that the SEM images (Figure 4 and Figure 5a–c) of [BmimCl]: butyramide indicate smaller wear scars compared with those of [BmimCl]: propionamide and [BmimCl]: propionamide. Abrasive grains can also be found in the wear surfaces lubricated with [BmimCl]: butyramide (Figure 5a–c). On the other hand, more abrasive grains can be found in the SEM images (Figure 5d) of [BmimCl]: butyramide (1:1), compared with those of [BmimCl]: butyramide (1:4, 1:6, 1:8) (Figure 4 and Figure 5e,f). Abrasive grains are produced due to the wear of the oxide film. More oxide films of [BmimCl]: butyramide (1:1) was generated due to its lower melting point, presenting a high supercooled liquid state.

**Figure 6:**
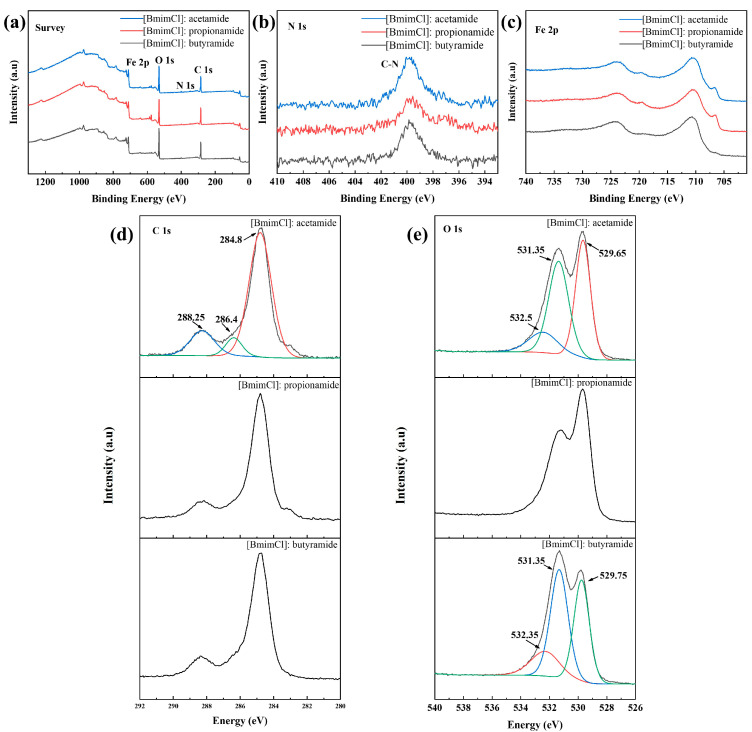
The XPS spectra of the worn surface lubricated with [BmimCl]: acetamide (1:4), [BmimCl]: propionamide (1:4), and [BmimCl]: butyramide (1:4): (**a**) survey, (**b**) N 1s, (**c**) Fe 2p, (**d**) C 1s, and (**e**) O 1s.

An XPS analysis was employed to get more details of the tribe film lubricated with [BmimCl]: acetamide (1:4), [BmimCl]: propionamide (1:4), and [BmimCl]: butyramide (1:4), as shown in Figure 6. The full XPS spectra (Figure 6a) of these DESs were similar. The peaks that should be observed are Fe 2p, O 1s, N 1s, and C 1s. The N element was detected in the XPS full spectra (Figure 6a) and the C-N peak was recorded at a binding energy of 399.71 eV (Figure 6b) [48]. This suggests that amides act as lubricants during friction. On the other hand, the peaks located at 288.25 eV was detected in C 1s spectrum (Figure 6d), which was the C=O peak of the amide. This indicates that the amide was adsorbed on the surface and showed lubrication under friction. For O 1s, there were peaks located at 532.5 eV, 531.35 eV, and 529.65 eV (Figure 6e), which are attributed to the results of tribo-chemical reactions (of FeO and Fe_2_O_3_) [49]. Interestingly, the O 1s peak of [BmimCl]: butyramide (1:4) is perfectly fitted via convolution, although the peak shape of [BmimCl]: butyramide (1:4) is different from that of [BmimCl]: acetamide (1:4) and [BmimCl]: propionamide (1:4). The results show that abrasive wear and oxidation wear occur on the surface of the friction trace under the action of these DESs.

### 3.4. Lubrication Mechanism

**Figure 7:**
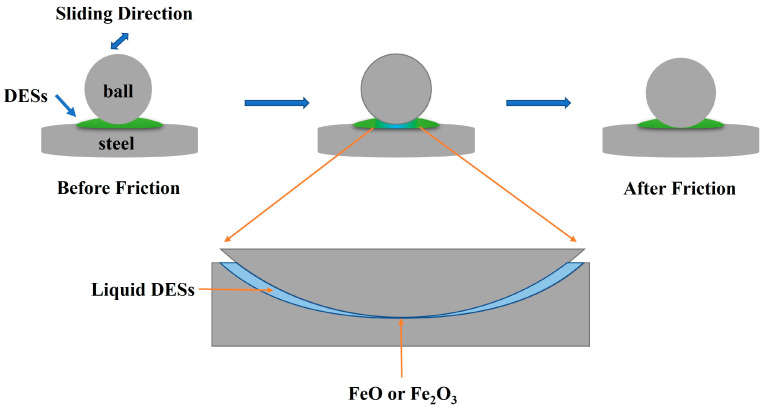
Related lubrication mechanism model of these DESs.

Based on the collected data above, a possible lubrication mechanism of these imidazole-based solid DESs at room temperature is illustrated in Figure 7. A friction ball mainly acts on the solid lubricant in the initiation of sliding, when friction is not yet stable. Friction tends to stabilize after a period of running. The heat generated around the friction changes the state of the solid lubricant due to the frictional heat effect. Deep eutectic solvents are changed from solid to liquid and a series of stable oxidizing chemical films are produced. These lubricant films play a role in reducing friction. The temperature is reduced to room temperature and the liquefied DES is converted into a solid state after the tribological test.

## 4. Conclusions

In this work, a series of imidazole-based DESs were prepared by shifting the eutectic point of the deep eutectic solvent. The lubricating properties of these solid DESs were evaluated on steel-to-steel friction pairs, and the most suitable lubrication mechanism was given. The relevant conclusions are as follows:

(1) These imidazole-based DESs behave as solids and semi-solids at room temperature. This allows them to undergo a lubricated state transition due to tribo-thermal effects.

(2) There is a positive relationship between the lubricating properties of these imidazole-based DESs and the alkyl chain length of the hydrogen bond donors. This wear volume is reduced from 30 × 10^6^ μm^3^ to 3 × 10^6^ μm^3^ approximately.

(3) The lubricating properties of DES are related to the content of hydrogen bond donors. The lubricating properties of [BmimCl]: butyramide (1:4) is more better than [BmimCl]: butyramide (1:1) due to the appropriate deviation of the eutectic point.

(4) This work provides an idea that takes advantage of DESs’ adjustable melting points to prepare solid lubricants. At the same time, DESs are expected to become an alternative to traditional cutting fluids.

## Figures and Tables

**Table 1:** List of prepared DESs.

DESs	Hydrogen Bond Acceptor	Hydrogen Bond Donors	Molar Ratio
[BmimCl]: acetamide	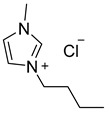	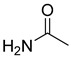	1:4
[BmimCl]: propionamide		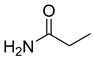	1:4
[BmimCl]: butyramide		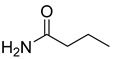	1:1
[BmimCl]: butyramide			1:4
[BmimCl]: butyramide			1:6
[BmimCl]: butyramide			1:8

**Table 2:** Table of freezing and melting points of [BmimCl]: acetamide, [BmimCl]: propionamide, and [BmimCl]: butyramide.

DESs	Freezing Point (°C)	Melting Point (°C)
[BmimCl]: acetamide (1:4)	7.45	30.24
[BmimCl]: propionamide (1:4)	9.78	44.10
[BmimCl]: butyramide (1:1)	24.56	44.19
[BmimCl]: butyramide (1:4)	40.72	93.01

**Table 3:** EDS table of all DESs.

DESs	Molar Ratio	Fe Element (wt%)	C Element (wt%)	O Element (wt%)
[BmimCl]: acetamide	1:4	68.11	3.75	0.59
[BmimCl]: propionamide	1:4	67.89	3.78	1.78
[BmimCl]: butyramide	1:1	69.06	2.67	1.32
[BmimCl]: butyramide	1:4	64.79	4.86	3.46
[BmimCl]: butyramide	1:6	65.93	3.48	2.00
[BmimCl]: butyramide	1:8	64.55	4.73	5.14

## Data Availability

The study did not report any data.
